# Operation mode of a step-feed anoxic/oxic process with distribution of carbon source from anaerobic zone on nutrient removal and microbial properties

**DOI:** 10.1038/s41598-018-37841-8

**Published:** 2019-02-04

**Authors:** Yijun Shen, Dianhai Yang, Yang Wu, Hao Zhang, Xinxi Zhang

**Affiliations:** 1 State Key Laboratory of Pollution Control and Resource Reuse, College of Environmental Science and Engineering, Tongji University, Shanghai, 200092 P.R. China; 2 Engineering Research Center of Biomembrane Water Purification and Utilization Technology, Ministry of Education, School of Civil Engineering and Architecture, Anhui University of Technology, Ma’anshan, 243032 P.R. China

## Abstract

This study investigated the operation mode of a step-feed anoxic/oxic (A/O) process with distribution of the carbon source from the anaerobic zone in terms of the treatment effects on sewage with low carbon and high nitrogen and phosphorus. After seven phases of operation, an optimal flow distribution ratio of 75%:25% was obtained from the anaerobic zone, and the concentrations of chemical oxygen demand, ammonia nitrogen, total nitrogen, and total phosphorous in the effluent were 20.8, 0.64, 14.2, and 0.89 mg/L, respectively. The presence of an internal reflux system in the deaeration zone improved the treatment. 16S rRNA high-throughput sequencing revealed that the microbial communities in aerobic zone I(O1) of the first-step A/O sludge were different from those in aerobic zone I (O2) of the second-step A/O sludge, whereas microbial communities of the seed sludge were similar to those in O2 of the second-step A/O sludge. The richness and diversity of microbial communities in O1 of the first-step A/O sludge samples were higher than those in O2 of the second-step A/O and seed sludge. At the optimal flow distribution ratio, the microbial abundance and treatment removal efficiency were the highest.

## Introduction

Eutrophication caused by high concentrations of nitrogen and phosphorus has serious effects on the environment and on human health globally. Chaohu Lake Basin, which is one of the five freshwater lakes in China, faces a serious eutrophication problem, and tail water from wastewater treatment plants (WWTPs) is one of the main sources of pollution. A previous study has indicated that nutrient pollution from effluents of wastewater plants is more serious than that from non-point sources^1^. Municipal wastewater in southern China is characterised by high nitrogen and phosphorus and low carbon content. Aiming at lower nitrogen and phosphorus concentrations is essential not only in the design of new plants, but also for upgrading existing wastewater treatment plants. The nitrogen and phosphorus removal efficiencies of conventional active sludge technologies are relatively low and are limited by the carbon source^2^. Several technologies, such as external organic carbon for post-denitrification and recirculation approaches, can be used to solve this problem. However, these countermeasures require additional resources and energy and hence, increase the operation costs and environmental footprint. Therefore, it is necessary to take full advantage of carbon sources in the raw wastewater. Optimal utilization of carbon sources is the main focus of step-feed technology, which optimizes carbon source distribution in the wastewater treatment process to rationally distribute limited carbon sources to denitrification and phosphorous removal reactors. Maximum utilization of carbon sources is achieved because invalid carbon oxidation in the aerobic zone is avoided^3–5^. Step-feed technology was first utilized in the 1990s and has since been implemented in China and other countries^6^.

The use of a three-stage step-feed anoxic/oxic (A/O) process for the treatment of raw wastewater resulted in reductions in the concentrations of total nitrogen (TN) and total phosphorus (TP) in the effluent from 20.8 to 14.2 mg/L and from 1.98 to 0.57 mg/L, respectively^7^. The treatment effect met the summer resource consent standard when the distribution ratio of a four-stage step-feed A/O reactor was set as 20%:30%:25%:25%^8^. A previous study reported effluent concentrations of chemical oxygen demand (COD), ammonia nitrogen ($N{H}_{4}^{+}$-N), TN, and TP of 33.05, 0.58, 9.26, and 0.46 mg/L, respectively, when the distribution ratio of a pilot modified step-feed A/O technology was set as 25%:35%:35%:10%^9^. An A/O-like two-stage biological aerated filter process, four-stage anaerobic/anoxic/oxic/oxic (A^2^/O^2^) process, and two-stage bioaugmented A/O biofilm process have been investigated^10–12^. The maximum $N{H}_{4}^{+}$-N removal reached 92.5%, 97.8%, and 95% when average influent concentrations were approximately 20.92, 228.2 ± 55.5, and 10–30 mg/L, respectively^10–12^.

Based on these previous studies, which mainly addressed the distribution of raw wastewater, we developed a novel step-feed A/O process with distribution of carbon sources from the anaerobic zone. We termed the process ‘anaerobic zone carbon source distribution (ACD) step-feed A/O’. In comparison to previously reported processes, we made two important adjustments to improve process performance: internal nitrification liquid recycling, and distribution of the carbon source from the anaerobic zone.

For efficient and steady operation of the ACD step-feed A/O process, it is imperative to understand the composition of microbial population in the process and to determine the relationship between reactor function and dominant population. Metagenomics allows analysing all microbes in an environmental sample without cultivation. It relies on high-throughput sequencing technology and efficient data processing software capable of managing large data volumes^13^. Metagenomics facilitates qualitative and quantitative descriptions of sub-dominant groups at relative abundances of 0.01–0.10%^14^. Microbial biodiversity and community structure are more accurately reflected by high-throughput sequencing because isolation and culture of microbes is not required^15^. The technology is increasingly used to precisely monitor bacterial communities in environmental samples, such as activated sludge^16,17^.

In this study, a pilot plant with a novel ACD step-feed A/O was utilised to treat domestic sewage and to investigate the effects of distribution ratio and reflux mode on denitrification and phosphorous removal capacities. High-throughput sequencing analysis was used to elucidate variations in the microbial community structure and succession of the dominant microbial communities. Control parameters of wastewater treatment in the pilot plant were optimized, and the relationship between reactor performance and the microbial community was determined. The results from this study are likely to contribute to our practical knowledge of and the theoretical basis for upgrading WWTPs.

## Results and Discussion

### Effect of flow distribution ratios on COD and nutrient removal efficiency

The test was run for 120 days in 7 phases. Phase I~VI was carbon source distribution of raw influent and the ratio was calculated by the flow feeding each in turn anoxic zone accounted for the proportion of the influent flow; while phase VII was carbon source distribution from anaerobic zone and the ratio was calculated by the flow feeding each in turn anoxic zone accounted for the proportion of the mix liquor flow (summation of the influent flow and returned activated sludge flow).The internal reflux was at the end of deaeration zone during phase I~III, whereas at end of aerobic zone I during phase IV~VII. The Flow distribution ratio was no distribution, 50%:50%, 25%:75%, 75%:25%, 75%:25%, 75%:25% and 75%:25% in each phase, respectively.

#### Effect of flow distribution ratio on COD removal efficiency

**Figure 1:**
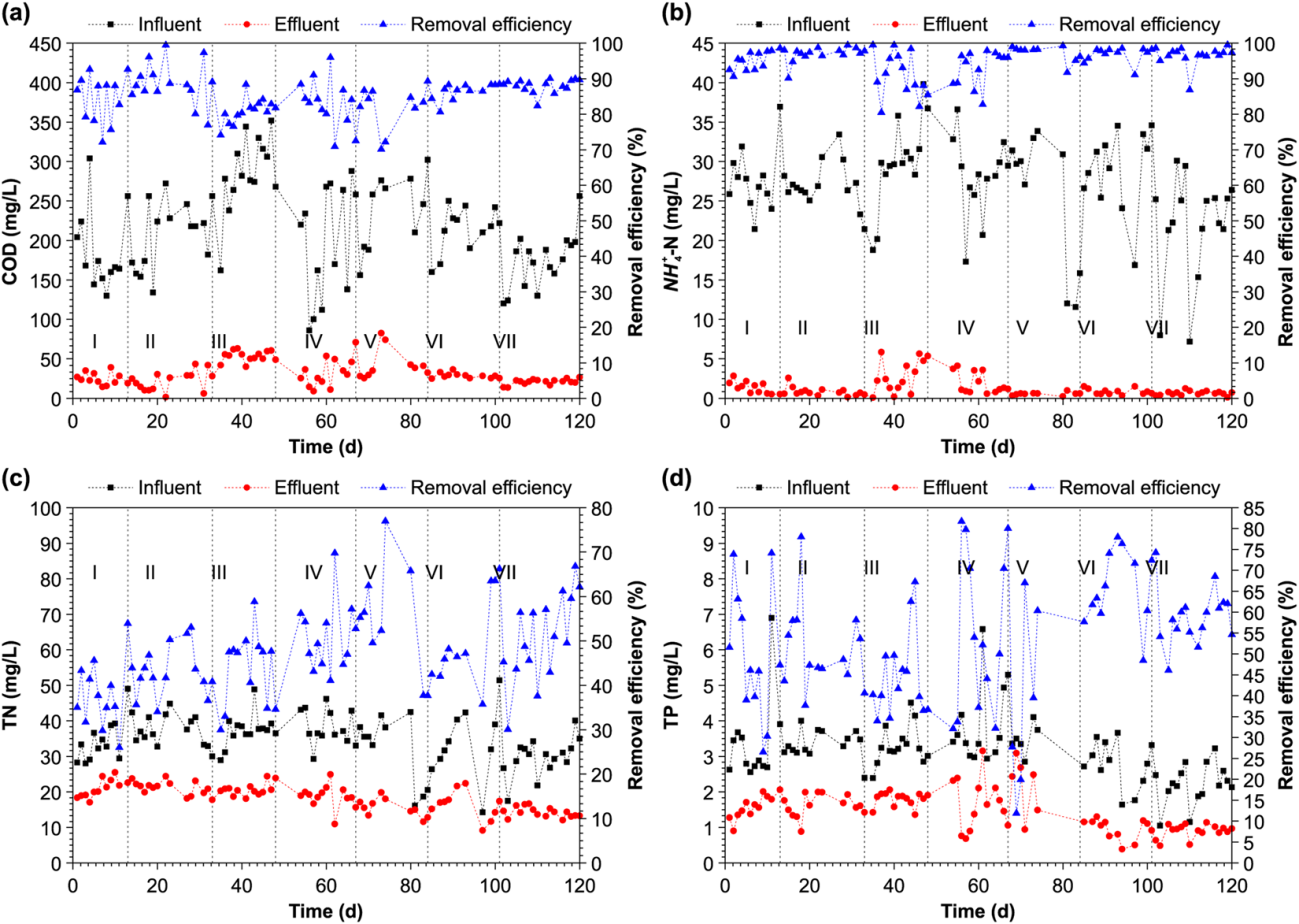
Effect of flow distribution ratios on removal efficiency for (**a**) COD, (**b**) $N{H}_{4}^{+}$-N, (**c**) TN, (**d**) TP.

COD removal by the pilot-scale reactor throughout the operation period is presented in Fig. 1a. The average COD concentrations in the effluents were 24.7, 21.7, 53.4, 32.9, 42.9, 28.7, and 20.8 mg/L, and the average removal efficiencies were 84.4%, 88.7%, 81.6%, 84.6%, 82.1%, 86.4%, and 88.0% for phases I to VII, respectively. The effluent concentrations in all phases except phase III met the integrated wastewater discharge standard (class A discharge standards, GB18918-2002, China; hereafter abbreviated as Class A). When the ratio of raw influent distribution was 25%:75% in phase III, the COD flow and total COD amount were high in aerobic zone II, very limited in the anaerobic zone and aerobic zone I, and the COD was inadequately depleted and was discharged with the effluent. The flow distribution ratio hardly had an effect on COD removal efficiency, and the removal capacity of the pilot-scale reactor was stable and efficient. As shown in Supplementary Fig. S1, the COD concentration along the flow tended to decrease in general. Nevertheless, the COD concentration in head of aerobic zone I (O1) to head of anoxic zone II (AN2) rose in phase VI, because 25% of the raw wastewater was distributed into anoxic zone II, and thus, COD in O1 was higher than that that in AN2. COD removal was higher in the anaerobic and anoxic zones than in the aerobic zone in all phases. This was because, as the raw wastewater flowed into the anaerobic and anoxic zones, the carbon source was utilised for the release of phosphorus and denitrification. Subsequently, the raw wastewater flowed into the aerobic zone, and the ineffective utilization of carbon source was averted and the competition among heterotrophic bacteria for the organic matter was reduced. These findings highlight the advantage of the distribution of carbon source from the anaerobic zone in the novel step-feed A/O technology.

#### Effect of flow distribution ratio on nitrogen removal efficiency

The average concentrations of effluent ammonia nitrogen ($N{H}_{4}^{+}$-N) were 1.37, 0.83, 2.80, 1.91, 0.56, 0.85, and 0.64 mg/L, and the average removal efficiencies were 95.0%, 96.9%, 91.0%, 93.1%, 97.1%, 96.8, and 96.5% from phases I to VII, respectively (Fig. 1b). The effluent quality of each phase satisfied the integrated wastewater discharge standard (Class A). The average concentrations of effluent TN were 21.0, 20.9, 19.3, 18.7, 15.4, 16.3, and 14.2 mg/L, and the average removal efficiencies were 37.8%, 43.1%, 47.4%, 50.8%, 50.1%, 50.1%, and 50.8% for phases I to VII, respectively (Fig. 1c). The effluent quality of phase VII satisfied the integrated wastewater discharge standard (Class A). These results indicate that the flow distribution ratio has a minimal influence on the removal of $N{H}_{4}^{+}$-N, but a strong influence on TN removal. As shown in Supplementary Fig. S2, the $N{H}_{4}^{+}$-N concentrations decreased along the flow; however, the trend in $N{H}_{4}^{+}$-N in phase IV was different from that in the other phases. A stirring paddle was installed between the anoxic zone and aerobic zone. Because of back mixing caused by unstable operation induced by the stirring paddle in phase IV, part of the flow at the beginning of anoxic zone II mixed with flow at the end of aerobic zone I, and therefore, part of the $N{H}_{4}^{+}$-N from the raw wastewater was diluted. In contrast, at the beginning of aerobic zone II, $N{H}_{4}^{+}$-N, which originated from the main stream of raw wastewater distribution, was not diluted; thus, the concentration of $N{H}_{4}^{+}$-N in aerobic zone I was higher than that in anoxic zone II. The decrease in $N{H}_{4}^{+}$-N was the most obvious in the anaerobic zone. First, because the $N{H}_{4}^{+}$-N was distributed into each reaction zone according the flow distribution ratio, it was diluted. Subsequently, $N{H}_{4}^{+}$-N was continuously diluted by the return sludge to result in obviously decreased concentrations. The concentration of effluent $N{H}_{4}^{+}$-N was generally below 2 mg/L (Fig. 1b), the nitrification capacity of the system was sufficient as indicated by effective nitrification; therefore, the removal effect of TN was determined by denitrification. As shown in Supplementary Fig. S3, TN removal was low due to insufficient carbon source in the anoxic zone I during phases II and III and, therefore, denitrification was inefficient and resulted in substantial accumulation of nitrate nitrogen $(N{O}_{3}^{-}$-N), which further lead to sub-optimal removal performance in subsequent reaction zone. The preferable treatment effect observed in phases VI and VII was due to the presence of sufficient carbon source in the anoxic zone I of first-step A/O. Therefore, denitrification was complete. Meanwhile, the internal reflux from aerobic zone I led to slight accumulation of nitrate nitrogen in anoxic zone I. The denitrification reaction was partly restrained by insufficient carbon source in anoxic zone II during phase VI. Because the carbon source was distributed from the anaerobic zone in phase VII, the denitrification reaction could proceed with more sufficient carbon source in anoxic zone II. The concentration of effluent nitrate nitrogen was approximately 9 mg/L, and TN removal was better than that during phase VII. The $N{O}_{3}^{-}$*-*N concentration decreased in the deaeration zone during phases V and VII, which was deduced to be the effect of endogenous denitrification. Microbial substances were used as carbon source for denitrification, which not only reduced the carbon source demand, but also reduced the sludge yield.

#### Effect of flow distribution ratio on phosphorus removal efficiency

The average concentrations of effluent TP were 1.58, 1.67, 1.68, 1.67, 2.18, 0.90 and 0.89 mg/L, and the average removal efficiencies were 40.7%, 48.1%, 50.4%, 56.0%, 37.7%, 67.3%, and 58.9% for phases I to VII, respectively (Fig. 1d). The amount of TP removed in the anaerobic zone during all phases except V was 34.0% (mass fraction), 21.6%, 27.6%, 28.0%, 15.7%, and 13.9%, respectively. Because the $N{O}_{3}^{-}$-N concentration of the returned activated sludge was relatively high, and the denitrifying bacteria had precedence over the polyphosphate-accumulating bacteria in utilizing the carbon source of the raw wastewater, the amount of carbon for anaerobic phosphorus release was reduced. Consequently, anaerobic phosphorus release was suppressed, which further influenced phosphorus removal. TP removal was the most efficient in phases VI and VII, which was deduced to result from lower denitrification. The relatively low temperature might have been one of the factors influencing TP removal, because it might have reduced the activity of polyphosphate-accumulating bacteria. Additionally, the maximum phosphate release rate as well as the maximum phosphorus uptake rate were reduced, thereby inhibiting phosphorus removal^18^. In previous related studies, phosphorus removal was effectively improved when the sludge retention time (SRT) was reduced and was controlled in 8 to 12 days^19^. On the one hand, activated sludge had higher biological activity and the bacteria contained more phosphorus in the case of a shorter SRT. On the other hand, polyphosphate-accumulating organisms (PAOs) are a class of heterotrophic microorganisms working optimally under short SRT, and phosphorus is removed by discharge of the phosphorus-rich excess sludge. Therefore, phosphorus removal is better when the SRT is short and the discharge amount of excess sludge is large. Because the SRT in the reactor process in this study was more than 20 days, phosphorus removal was not satisfactory. General trends in phosphate concentrations in different phases along the flow are shown in Supplementary Fig. S4. The reduction in phosphate was the most obvious in the anaerobic zone. In general, the most efficient phosphorus removal was observed during phases VI and VII, in which sufficient carbon was present to allow anaerobic phosphorus release in the anaerobic zone. The aerobic phosphorus uptake increases with an increase in anaerobic phosphorus release; therefore, the effect on TP removal could be improved^20,21^. In these phases, the maximum phosphorus release rate was 448%, which is in accordance with rates reported in other studies, i.e., 415%^9^ and 495%^22^. Therefore, TP removal could be improved by adjusting process parameters, such as the flow distribution ratio and the internal reflux ratio, while maintaining a lower concentration of nitrate nitrogen in the anaerobic zone and ensuring optimal TN removal. As shown in Supplementary Fig. S4, TP removal occurred to a certain extent in the anoxic zone of each phase, and it is concluded that denitrifying phosphate-accumulating organisms bacteria used nitrate nitrogen as electron acceptor for denitrification phosphorus removal, which was most efficient when the flow distribution ratio was 75%:25%.

### Effect of internal reflux position on pollutant removal efficiency

**Figure 2:**
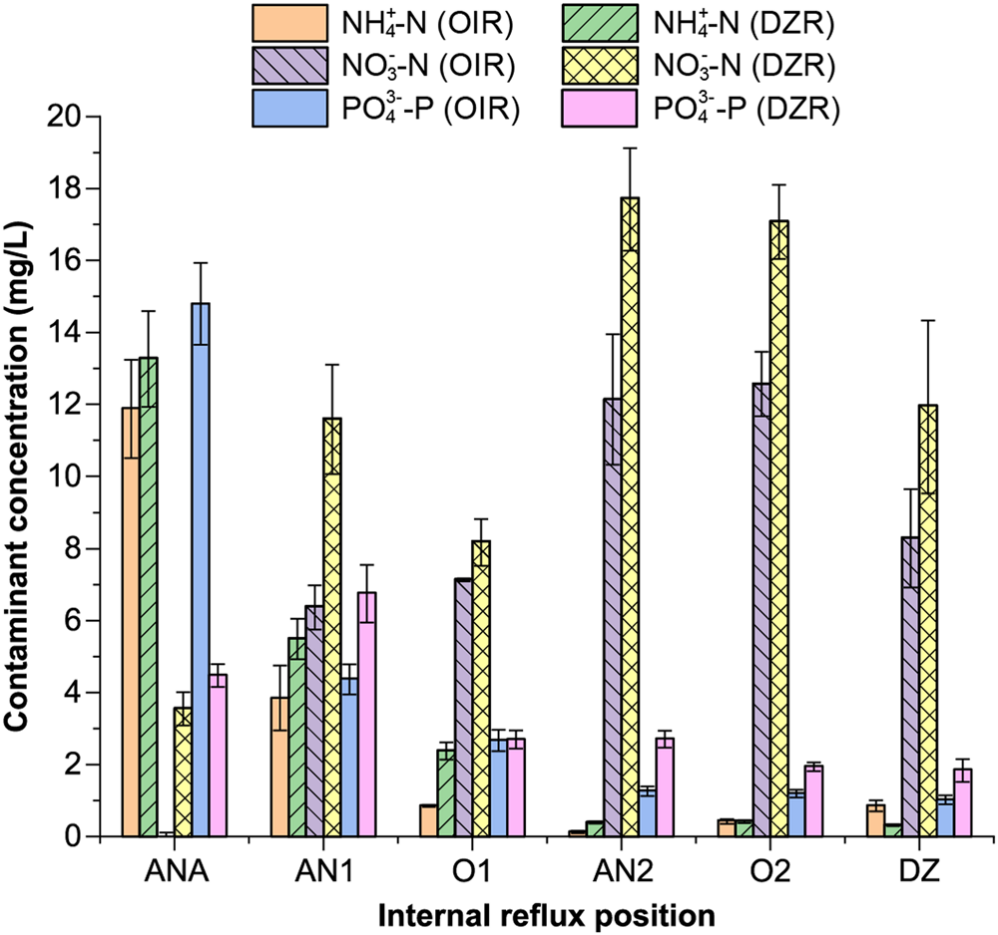
Effect of different internal reflux system position on contaminate concentration. OIR indicates that the internal reflux system was positioned at the end of aerobic zone I. DZR indicates that the internal reflux system was positioned at the end of the deaeration zone.

In this study, the internal reflux port was located at the end of deaeration zone (DZR) during phases I, II, and III and at the end of aerobic zone I (OIR) during phases IV, V, VI, and VII. The concentrations of ammonium, nitrate and phosphate along the flow were measured to investigate the effect of the position of internal reflux. The nitrification liquor containing abundant nitrate nitrogen was recycled into anoxic zone I by the internal reflux system to utilise nitrate nitrogen as an electron acceptor for denitrification in the anoxic zone. As shown in Fig. 2, the position of the internal reflux system had no significant impact on the efficiency of ammonium nitrogen removal and ammonium nitrogen concentrations along the flow. However, denitrification was better when the internal reflux system was positioned at the end of the deaeration zone. The relatively poor denitrification when the internal reflux system was positioned at the end of aerobic zone I was due to insufficient nitrate nitrogen in the mixed liquor, which was the electron acceptor for denitrification. The concentration of phosphate phosphorus declined in the anoxic zone (special anoxic zone I) due to the dilution of mixed liquor and denitrifying phosphorus removal. The decline in phosphate was larger when the internal reflux system was positioned at the end of the deaeration zone than when it was positioned at the end of aerobic zone I. Therefore, placement of the internal reflux system at the end of the deaeration zone promoted phosphorus removal.

### Molecular biological characteristics of sludge at various phases

High-throughput sequencing was used to assess microbial diversity and community structure of the seed sludge and sludge samples taken at phases V(sample 150609ZS1,150609ZS2), VI(sample 150714ZS1,150714ZS2), and VII (sample 150803ZS1,150803ZS2). Suffix named ZS1 represented samples taken from aerobic zone I(O1), and suffix named ZS2 represented samples taken from aerobic II(O2).The sludge samples were obtained from the aerobic zone because microorganisms in this zone are the most representative^23^. Alpha diversity indices for microbial community distribution are listed in Table 1. The coverage index indicates the percentage of individuals sampled in the microbial community. The coverage indices ranged from 71.0% to 89.2% and represented the main bacterial species in the samples, indicating that the sample was representative of the microbial community in the sludge. The abundance-based coverage estimation (ACE) index and Chao1 index (Table 1) give an indication of species richness. Samples of aerobic zone II of the pilot-scale reactor had a similar richness, which ranged from 40000 to 50000 (ACE) in different phases, and richness in the seed sludge ranged from 15000 to 30000 (ACE), which was similar to that in aerobic zone II. The microbial richness in aerobic zone I was higher than that in aerobic zone II and the seed sludge. It can be concluded that the step-A/O process with carbon source distributed from the anaerobic zone is more favourable for microbial richness than the A^2^/O technology of the local WWTP where the seed sludge was obtained from. In aerobic zone I, there was more organic matter and nutrition for the bacteria to grow. Additionally, the main biochemical reactions in aerobic zone I were nitrification and phosphorus accumulation, which resulted in a higher microbial richness this zone.

**Table 1:** Alpha diversity of activated sludge samples.

Phase	Sample_ID	Sequences	OTUs	ACE^a^	Chao1^b^	Shannon^c^	Simpson^d^	Coverage^e^
Seed sludge	150610WXY	23896	5544	27899	16027	7.26	0.00281	0.848008
V	150609ZS1	20036	7970	48117	25703	7.87	0.00250	0.710421
	150609ZS2	23129	4314	14646	10028	7.08	0.00263	0.892473
VI	150714ZS1	17875	7401	42624	23752	7.91	0.00202	0.700196
	150714ZS2	21806	5313	22784	14193	7.26	0.00326	0.845364
VII	150803ZS1	18482	7306	46552	23615	7.78	0.00249	0.710908
	150803ZS2	22910	5685	29823	17130	7.27	0.00370	0.834745

^a,b^Community richness (a higher number represents greater richness).

^c,d^Community diversity (a higher Shannon index represents greater diversity while a higher Simpson value represents lower diversity).

^e^Sampling depth.

Shannon and Simpson indices give an indication of microbial community biodiversity. A higher Shannon index represents greater diversity, whereas a higher Simpson index indicates lower diversity. As shown in Table 1, trends in these indices were quite consistent with those in ACE and Chao1. The biodiversity in aerobic zone I was higher than that in aerobic zone II and the seed sludge. Moreover, the biodiversity in aerobic zone I was nearly equal in the three different phases, and the same as that in aerobic zone II. The biodiversity in the seed sludge was nearly equal to that in aerobic zone II. Microbial community biodiversity affects system stability; a high biodiversity indicates that the community consists of populations with different biological and ecological features, resulting in greater stability and resistance to volatility^24^. The activated sludge system is a mini ecosystem, and certain functions of the reactor are enhanced as certain microorganisms decrease and other, functional bacteria increase in systems such as nitritation^25–27^, phosphorus removal^28^, and anammox^29^ systems. Stabilization of the activated sludge system is based on the accumulation of process-functional bacteria, whereas has limited relevance with microbial community diversity. The accumulation of functional bacteria mainly relies on the artificial reinforcement in the system. Therefore, sample biodiversity did not obviously differ among different phases.

### Microbial community composition of sludge at various phases

**Figure 3:**
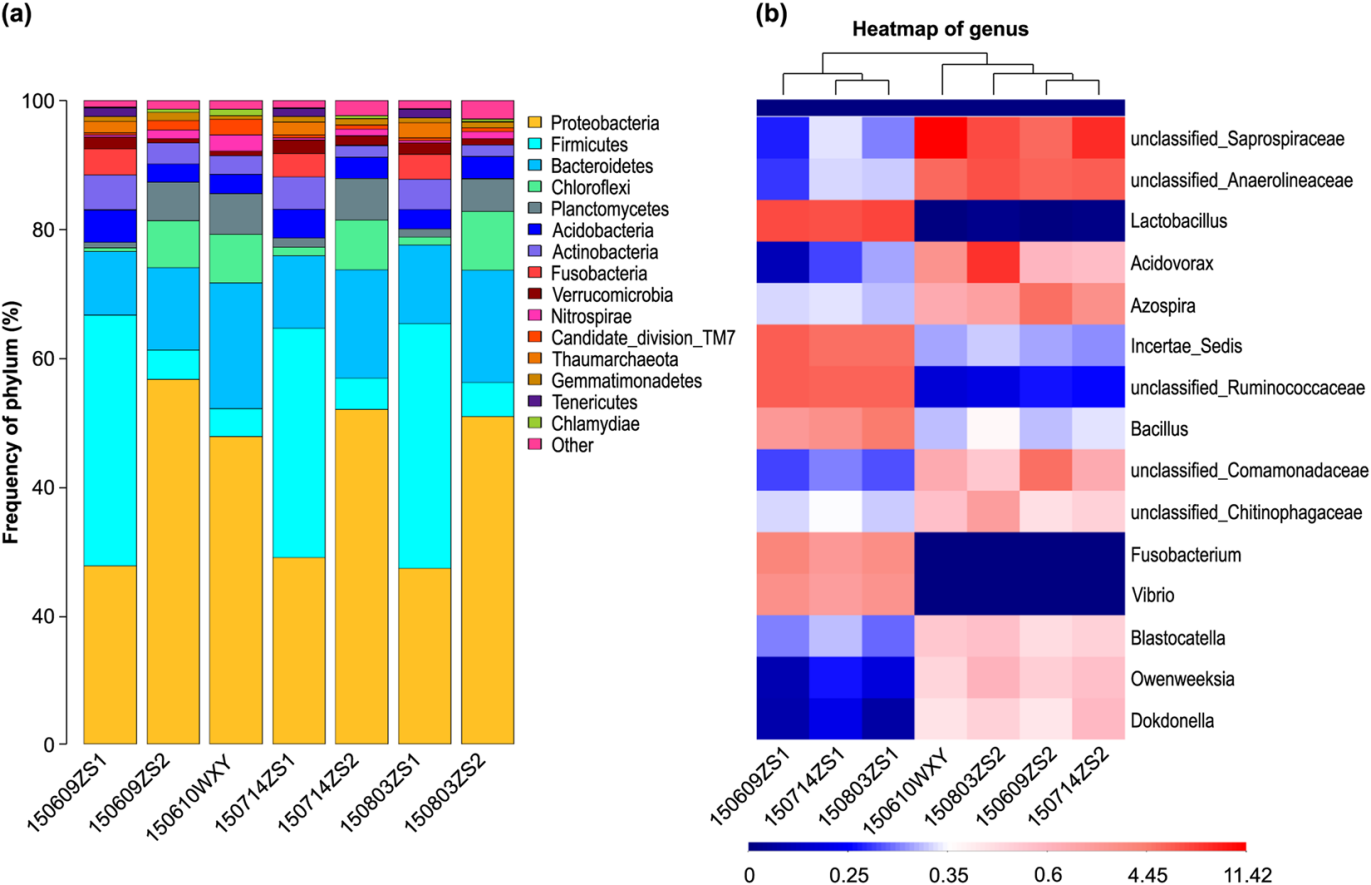
Relative abundances of the microbial communities at (**a**) phylum level and (**b**) genus level in the three different phases and seed sludge. Less than 1% of total the bacterial community composition was classified as ‘others’.

Figure 3 shows the dynamic changes in community composition in terms of relative abundances of different phyla (Fig. 3a) and genera (Fig. 3b) in the three phases samples and the seed sludge. Non-metric multidimensional scaling (NMDS) (Fig. S5) could effectively evaluate the similarity and difference of microbial communities. *Proteobacteria* were predominant in the seed sludge, followed by *Bacteroidetes*, *Chloroflexi* and *Planctomycetes*. *Firmicutes* were predominant in aerobic zone I, followed by *Proteobacteria*, *Bacteroidetes* and *Actinobacteria*. *Proteobacteria* were predominant in aerobic zone II, followed by *Bacteroidetes*, *Chloroflexi* and *Planctomycetes*. The relative abundances of *Acidobacteria*, *Fusobacteria*, *Verrucomicrobia*, *Thaumarchaeota*, and *Tenericutes* in aerobic zone I and *Firmicutes*, *Acidobacteria*, *Actinobacteria*, and *Nitrospirae* in aerobic zone II and seed sludge were higher than 1%. As shown in Fig. 3a, the microbial community of the seed sludge was similar to that of aerobic zone II, but different from that of aerobic zone I. These results are consistent with those of a previous study^30^. The differences among the seven samples were apparent at the genus level, especially for samples in aerobic zones I between samples in aerobic zones II and the seed sludge. In total, there were 444 (150609ZS1), 391 (150609ZS2), 384 (150610WXY), 638 (150714ZS1), 399 (150714ZS2), 622 (150803ZS1), and 407 (150803ZS2) genera in the seven samples, respectively. Additionally, 22 (150609ZS1), 25 (150609ZS2), 23 (150610WXY), 26 (150714ZS1), 21 (150714ZS2), 23 (150803ZS1), and 20 (150803ZS2) genera had an abundance greater than 1% in all samples. The number of genera in aerobic zone I was nearly twice that in aerobic zone II, especially in sludge samples from the last two phases of the pilot-scale reactor. A heatmap (Fig. 3b) for the 15 most abundant genera was constructed to evaluate similarities and differences among the seven samples. Based on the heatmap, the seven samples could be divided into two categories, i.e., three samples in aerobic zone I that had similar composition, and the seed sludge microbial community, which had a composition similar to that in the other three samples in aerobic zone II. This view was also confirmed by NMDS diagrams (Fig. S5). *Lactobacillus*, Incertae Sedis, unclassified *Ruminococcaceae*, *Fusobacterium* and *Vibrio* were the five most dominant genera in aerobic zone I. Unclassified *Saprospiraceae*, unclassified *Anaerolineaceae*, *Acidovorax*, *Azospira* and unclassified *Comamonadaceae* were the five most dominant genera in aerobic zone II and the seed sludge. Because the supplement sludge of phase V was taken from the seed sludge (150610WXY), the main genera of samples (150714ZS2, 150803ZS2) were similar to those in the seed sludge, especially for 150714ZS2 (phase VI). In phase VI (150610WXY, 150714ZS2), the dominant genus was unclassified *Saprospiraceae*, whereas in the last phase, where carbon source distribution was from the anaerobic zone, the dominant genus was *Acidovorax* (150803ZS2). The sludge samples from before the addition of supplement sludge (150609ZS2) were different from the seed sludge in terms of composition, and unclassified *Anaerolineaceae* was the dominant genus. *Lactobacillus* is a class of acid-resistant chemotrophic bacteria that can produce lactic acid from carbohydrate. Therefore, more than 50% the final product was lactic acid fermented by *Lactobacillus* which is widely used in sewage or sludge treatment^31^. Unclassified *Saprospiraceae* are abundant in enhanced biological phosphorus removal plants and in anaerobic digestion^32^. *Anaerolineaceae* are often abundant in sludge from mainland China^16^ and their main function is to degrade carbohydrates or cell tissues in the anaerobic system^33^.

### Dynamic changes in functional microorganisms

**Figure 4:**
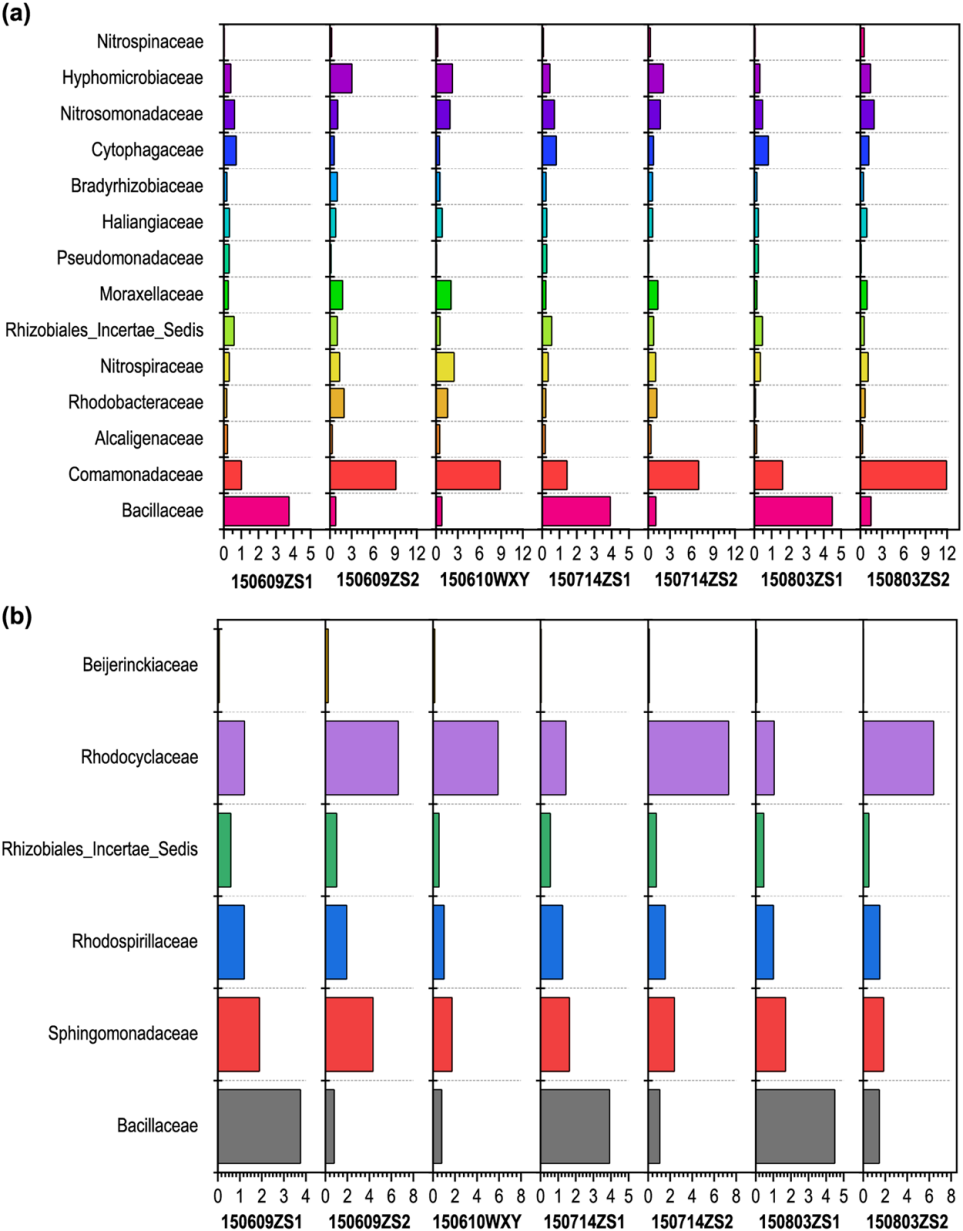
Abundances of potential (**a**) nitrifying and denitrifying bacteria and (**b**) phosphorus-removing bacteria in the seven samples.

Nitrifying and denitrifying bacterial communities play an important role in ammonia removal in the step-A/O with carbon resource distribution from the anaerobic zone. Ammonia-oxidizing bacteria (AOB) and nitrite-oxidizing bacteria (NOB) collectively convert ammonium to nitrate during the nitrification process, and TN is removed during the denitrification process. AOB can be classified into β and γ subclasses of *Proteobacteria* according to a previous study^34^. The main AOB genera are *Nitrosomonas*, *Nitrosococcus*, *Nitrosospira*, *Nitrosolobus*, and *Nitrosovibrio*^35^. *Nitrospira*, *Nitrospina*, *Nitrobacter*, and *Nitrococcus* are core NOB. *Nitrospira* belong to the phylum *Nitrospirae* and the other NOB have been classified into three subclasses of *Proteobacteria* and *Chloroflexi*^36^. In the seven sludge samples, AOB detected belonged to unclassified *Nitrosomonadaceae*, other AOB genera were not found. The AOB abundances in aerobic zones I and II were 0.48–0.7% and 1.05–1.89% (Fig. 4a), respectively. The AOB abundance in the seed sludge was 1.91%. The NOB genera detected in the seven samples were *Nitrospira*, *Nitrospina*, and *Nitrobacter*, whereas *Nitrococcus* was not detected. *Nitrospira* was the most abundant genus, with an abundance of more than 0.25% in aerobic zone I and of 0.98% in the other samples. *Nitrospina* and *Nitrobacter* were substantially less abundant than *Nitrospira*. Thus, it is concluded that *Nitrosomonas* and *Nitrospira* were the dominant genera in the AOB and NOB communities, respectively. As shown in Fig. 4a, the AOB abundance in aerobic zone II was nearly same as that in the seed sludge, but 2–3 times that in aerobic zone I. The total abundance of AOB in the pilot-scale reactor was greater than that in the seed sludge. The NOB abundance in aerobic zone II was 3–4 times that in aerobic zone I, and the total NOB abundance in the pilot-scale reactor was a slightly lower than that in the seed sludge. Nitrification mainly occurred in aerobic zone II; therefore, the presence of an internal reflux system in the deaeration zone was beneficial for the removal of TN. The total AOB abundance in aerobic zones I and II increased with each reaction phase; however, the total NOB abundance in these zones remained quite constant. It is inferred from Figs 1c and 4a that the 75%:25% distribution from the anaerobic zone was favourable for nitrification. Denitrification capacity is widely found in bacteria, archaea, and some eukaryotes (e.g., fungi), and bacteria are primarily responsible for nitrate reduction in natural and engineered ecosystems^17^. Denitrification capacity is widespread among bacteria, which are taxonomically and phylogenetically diverse. The potential denitrifying bacteria in the seven samples were classified into 11 families (Fig. 4a). The denitrifying bacterial families in three samples from aerobic zone I had very high similarity because the denitrifying bacteria come from anoxic zone I, where not only were the carbon source and nitrate of reflux liquid sufficient, but the environment was consistent and suitable for bacterial sustenance. The first six families were the same, i.e., *Bacillaceae* (3.75–4.49%), *Comamonadaceae* (1.01–1.62%), *Cytophagaceae* (0.71–0.81%), *Rhizobiales* Incertae Sedis (0.46–0.59%), *Hyphomicrobiaceae* (0.32–0.45%) and *Haliangiaceae* (0.2–0.32%). *Bacillaceae* and *Comamonadaceae* were the dominant denitrifying bacteria and their combined abundance was approximately 60% of the total abundance of denitrifying bacteria in aerobic zone I, with *Bacillaceae* accounting for 45% of the total abundance. The denitrifying bacterial families in three samples from aerobic zone II were different each other in the three phases. Only the most abundant denitrifying bacteria, *Comamonadaceae*, which accounted for 40–50% of the total denitrifying bacterial abundance in aerobic zone II, was similar. The concentration of nitrate was not very high in anoxic zone II of the second step of A/O and the carbon resource was relatively insufficient; therefore, the variation in the denitrifying bacterial community could be attributed to substrate diversification. The bacterial families in aerobic zone II during phase VI were quite similar to those in the seed sludge, whereas families in phase VII were different from those in the seed sludge. The total abundance of denitrifying bacteria was higher in phase VII than in phases VI and V, which is consistent with the fact that the TN removal efficiency was the highest during phase VII (Fig. 1c). *Comamonadaceae* are typical anoxic denitrifying bacteria and have also been reported by Spring *et al*.^37^ and Jahan *et al*.^38^. *Bacillus* (family *Bacillaceae*) are aerobic denitrifying bacteria that can convert $N{O}_{3}^{-}$-N to N_2_ in an aerobic environment^39^. In general, both anoxic denitrifying bacteria and aerobic denitrifying bacteria are responsible for TN removal. The dominant microorganisms associated with phosphorus removal are shown in Fig. 4b. *Beijerinckiaceae*, *Bacillaceae*, *Sphingomonadaceae*, *Rhizobiales* Incertae Sedis, *Rhodospirillaceae*, and *Rhodocyclaceae* were the six families constituting the core PAOs. The PAO community in the first and second step of A/O was quite similar in the three phases, and the PAO community in the seed sludge was highly similar to that in the second step of A/O of the pilot-scale reactor. *Bacillaceae* was the most abundant family in the first-step A/O, and its abundance in each phase was 3.75%, 3.93%, and 4.49%, respectively. *Rhodocyclaceae* was the most abundant family in the second-step A/O, and its abundance in each phase and in the seed sludge was 6.64%, 7.32%, 6.4%, and 5.92%, respectively. Previous studies have found uncultured *Rhodocyclaceae* to be dominant in the active sludge of the enhanced biological phosphorus removal reactors^40,41^. *Rhodospirillaceae* have been reported to be PAOs^42^; however, another study argued that *Rhodospirillaceae* are glycogen-accumulating organisms^43^. As shown in Fig. S4, denitrifying phosphorus removal was observed, which is consistent with other studies that have found *Bacillaceae*, *Rhodocyclaceae*, and *Hyphomicrobiaceae* to be typical denitrifying PAOs^44^. The total abundance of PAOs was the highest in phase VII among the three phases; therefore, the efficiency of phosphorus removal was also the highest in this phase.

## Disscusion

In this study, the ACD step-feed A/O process was applied to treat sewage with low carbon source and high nitrogen and phosphorus. The optimal flow distribution ratio obtained from the anaerobic zone was 75%:25% and the effluent concentration was consistent with the class A discharge standards. The distribution ratio had negligible influence on the removal of COD and $N{H}_{4}^{+}$-N but had a large influence on the removal of TN and TP. The internal reflux position in the deaeration zone improved the removal of TN and TP. High-throughput sequencing revealed the pollutant removal mechanisms, microbial communities and functional microbial characteristics in the seven sludge samples. The microbial communities in O1 sludge was highly different from those in O2 while microbial communities of seed sludge was very similar to those of O2 sludge in the pilot plant. The abundances of corresponding major functional species were the highest in the pilot-scale plant when the operation mode was optimal and *Nitrosomonas*, *Nitrospira*, *Bacillaceae* and *Rhodocyclaceae* were the dominant nitrogen and phosphorus removal communities, respectively. In general, this process offers several advantages proved by the test. First, when the flow distribution is from the anaerobic zone, the carbon sources in raw wastewater can be preferentially utilized by phosphorus-accumulating organisms to enhance phosphorus removal. Second, slow degradation of COD can be converted into the solution COD in the anaerobic zone, therefore, the quality of carbon sources can be improved; finally, the sludge from the anaerobic zone can also be used as carbon sources by the microbes.

## Methods

### ACD step-feed A/O pilot reactor

**Figure 5:**
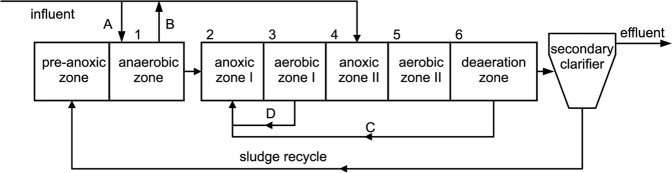
Schematic of the ACD Step-feed A/O. 1–6 indicate the measure point positions of variation along the flow. (1) anaerobic zone (ANA), (2) head of anoxic zone I (AN1), (3) head of aerobic zone I (O1), (4) head of anoxic zone II (AN2), (5) head of aerobic zone II (O2), (6) deaeration zone (DZ). (**A**) Distribution of raw influent; (**B**) Distribution from the anaerobic zone; (**C**) Internal reflux position in phases I, II, III; (**D**) Internal reflux position in phases IV, V, VI, VII. The distribution of raw influent was not simultaneous with the distribution from the anaerobic zone.

In this study, domestic wastewater was treated in a novel step-feed A/O with distribution of carbon sources from the anaerobic zone of the pilot plant. A schematic is presented in Fig. 5. The volume ratio of the pre-anoxic, anaerobic, anoxic, aerobic and deaeration zones was 1.0:4.0:4.8:6.5:2.6, and the total hydraulic retention time was 17 h.

### Seed sludge and wastewater

Raw wastewater was obtained from the effluent of a vortex-type grit chamber of a WWTP in Hefei, China. The average C/N (mass) and C/P (mass) ratios were 6.2 and 70, respectively (Table 2), thus, the organic carbon source was typically limiting^45^. Seed sludge was also obtained from the secondary clarifier of the WWTP. The performance of the sludge was satisfactory after being domesticated and cultured for 15 days.

**Table 2:** Quality of the raw water.

Content	Range	Average
COD (mg/L)	52–352	205
${\rm{N}}{{\rm{H}}}_{4}^{+}$-N (mg/L)	7.2–39.8	27.1
${\rm{N}}{{\rm{O}}}_{2}^{-}$-N (mg/L)	0.0534–0.550	0.156
${\rm{N}}{{\rm{O}}}_{3}^{-}$-N (mg/L)	0.0–17.3	1.0
TN (mg/L)	14.2–49.0	34.5
${\rm{P}}{{\rm{O}}}_{4}^{3-}$ -P (mg/L)	0.6–7.8	2.5
TP (mg/L)	1.04–4.93	3.06

### Parameters tested and analytical methods

COD, TP, phosphate ($P{O}_{4}^{3-}$-P), $N{H}_{4}^{+}$-N, nitrite ($N{O}_{2}^{-}$-N), $N{O}_{3}^{-}$-N, TN, mixed liquor suspended solid (MLSS), and mixed liquor volatile suspended solid (MLVSS) were measured using standard methods^46^. Dissolved oxygen (DO), oxidation-reduction potential (ORP), and temperature were measured continuously by on-line probes (HACH, USA), and the pH was measured with a HQ40d portal multi meter (HACH).

### Experimental procedure

The pilot-scale reactor test lasted for 120 days and was conducted at a temperature of 10–25 °C. The influent flow, internal reflux ratio, and returned activated sludge ratio were set as 2 m^3^·h^−1^, 200%, and 50%, respectively. The concentration of MLSS was 3000–4500 mg/L and was controlled by discharging excess sludge from the bottom of the secondary clarifier. A DO probe was placed in the aerobic zones where air was pumped from the disc aerator at the bottom of the reactor, and DO concentrations were automatically controlled at 2.0 mg/L. The test procedure was divided into seven phases, and the pollutant removal rate, which was affected by the flow distribution ratio, was analysed during different phases, while other reaction conditions were kept constant. Running modes and control parameters in the different phases are shown in Table 3. Sludge was supplied to the pilot-scale reactor in phases V, VI, and VII to maintain the MLSS concentration. The effect of pollution variation along the flow was tested in the last few days of each stable operation phase.

**Table 3:** Operation modes and control parameters in different phases.

Phase	Time (days)	Flow distribution ratio^a^	Sludge retention time (days)	Position of the internal reflux
I	1–13	no distribution of the raw influent	15–20	end of deaeration zone
II	14–33	ID 50%:50%	15–20	end of deaeration zone
III	34–53	ID 25%:75%	15–20	end of deaeration zone
IV	54–67	ID 75%:25%	no sludge discharge	end of aerobic zone I
V^b^	68–84	ID 75%:25%	no sludge discharge	end of aerobic zone I
VI	85–101	ID 75%:25%	25–30	end of aerobic zone I
VII	102–120	AD 75%:25%	25–30	end of aerobic zone I

^a^ID was carbon resource distribution of raw influent, and the ratio was calculated by the flow feeding each in turn anoxic zone accounted for the proportion of the influent flow; AD was carbon resource distribution from the anaerobic zone, and the ratio was calculated by the flow feeding each in turn anoxic zone accounted for the proportion of the mix liquor flow (summation of the influent flow and returned activated sludge flow).

^b^The pollution variation along the flow was not measured during phase V because the sludge of this phase was cultured after the supplemental sludge was added in the pilot-scale reactor.

### Microbial community analysis

#### DNA extraction and PCR amplification

Sludge sample 150610WXY was taken from the seed sludge, while samples 150609ZS1, 150714ZS1, and 150803ZS1 were taken from aerobic zone I of the pilot reactor. Samples 150609ZS2, 150714ZS2, and 150803ZS2 were taken from aerobic zone II. 150609ZS1, 150609ZS2 represented phase V; 150714ZS1, 150714ZS2 represented phase VI and 150803ZS1, 150803ZS2 represented phase VII. The Samples from the pilot reactor was taken at the end of the each phase. Total active sludge DNA was extracted using the E.Z.N.A.® Soil DNA kit (Omega Bio-Tek, Inc., Norcross, GA, USA), and the DNA quality was assessed by 2% (w/v) agarose gel electrophoresis^47^. The V3–V4 regions of the bacterial 16S ribosomal RNA gene were amplified by PCR (BIO-RAD T100^TM^ Thermal Cycler, USA) with universal primers 341 F (5′-CCTACGGGNGGCWGCAG-3′) and 805 R (5′-GACTACHVGGGTATCTAATCC-3′). Details of the PCR procedure are available in the Supplementary Methods.

#### Illumina MiSeq sequencing

The DNA samples were paired-end sequenced (2 × 200 bp) on an Illumina MiSeq platform (Sangon Biotech, Shanghai, China) according to standard protocols. Primer sequences, reads shorter than 50 bp, and sequences with low complexity and quality were removed using the Prinseq software (Vision 0.20.4). Read quality met the basic analysis requirement because the length of most sequences was within 400–600 bp, the average length of sequences was 440 bp, and the number of sequences was more than 500 after quality control. Operational taxonomic units (OTUs) were clustered using a 97% similarity cut-off via Uclust (vision 1.1.579), and chimeric sequences were identified and removed using chimera.uchime (Mothur, Vision 1.30.1) based on alignment against the SILVA database (http://www.arb-silva.de/). The taxonomy of each 16S rRNA gene sequence was analysed with RDP Classifier (http://rdp.cme.msu.edu/) against the SILVA 16S rRNA database, using a confidence threshold of 80%. Sequence richness was calculated for each sample and species taxonomic units were determined to construct richness arrays of samples and species taxonomic units. Species diversity and community differences were determined by alpha diversity analysis. Richness and diversity of microbial communities were evaluated on the basis of ACE, Chao 1, Simpson, and Shannon indices using Mothur (v.1.30.1, http://www.mothur.org/).

## Supplementary information

Supplementary Information

## Data Availability

All data generated or analysed in this study are included in this published article and in the Supplementary Information files.
