# Supplementary material for: Operation mode of a step-feed anoxic/oxic process with distribution of carbon source from anaerobic zone on nutrient removal and microbial properties
Source: Sci Rep. 2019 Feb 4;9:1153. doi: 10.1038/s41598-018-37841-8 (PMC6362077; doi:10.1038/s41598-018-37841-8)
Supplement: Supplementary file 1 — Supplementary Information [file 41598_2018_37841_MOESM1_ESM.pdf]

Supplementary Information for:

Operation mode of a step-feed anoxic/oxic process with distribution of carbon source from anaerobic zone on nutrient removal and microbial properties

Yijun Shen [1,2], Dianhai Yang[1,*], Yang Wu [2], Hao Zhang[1] & Xinxi Zhang[2]

[1] State Key Laboratory of Pollution Control and Resource Reuse, College of Environmental Science and Engineering, Tongji University, Shanghai, 200092, P.R. China.
[2] Engineering Research Center of Biomembrane Water Purification and Utilization Technology, Ministry of Education, School of Civil Engineering and Architecture, Anhui University of Technology, Ma'anshan, 243032, P.R. China.

*Corresponding author: Dianhai Yang, Tel: +86-13501914870, Fax: +86-02165986313, E-mail: yangdianhai@tongji.edu.cn

This document consists of 6 pages, including Methods and 4 figures.

Fig. S1 Variation in COD concentrations along the flow in different phases.
Fig. S2 Variation in ammonia nitrogen concentration along the flow during different phases.
Fig. S3 Variation in nitrate nitrogen concentration along the flow during different phases.
Fig. S4 Variation in phosphate concentration along the flow during different phases.
Fig. S5 Community profiles of the samples based on nonmetric multidimensional scaling(NMDS).

Methods

Microbial community analysis

PCR amplification

PCRs were carried out in a 50-μl mixture containing 5 μl of 10× PCR buffer, 0.5 μl of 10 mM dNTPs, 10 ng of genomic DNA, 0.5 μl of 50 μM Bar-PCR primer F, 0.5 μl of 50 μM primer R, and 0.5 μl of 5U/μl Platinum Taq. Amplicons were extracted from agarose gels after electrophoresis and were purified using the SanPrep Column DNA Gel Extraction Kit (Sangon Biotech, Shanghai, China) and quantified on a Qubit 2.0 using a Qubit™ ssDNA Assay Kit (Thermo Fisher Scientific, Eugene, OR, USA). The purified amplicons were pooled in equimolar amounts, mixed thoroughly, and processed for (barcode-added) library preparation and sequencing on .

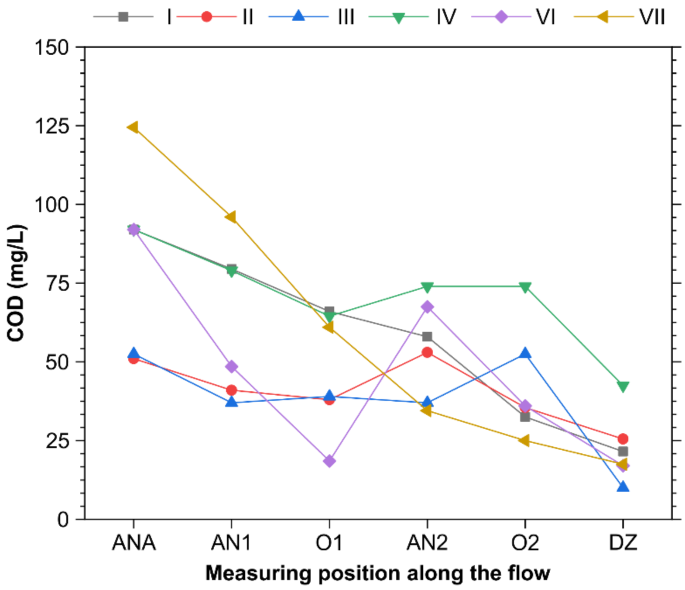

Supplementary Figure S1 Variation in COD concentration along the flow in different phases.
Note: Variation in pollution along the flow was not measured during phase V because the sludge in this phase was cultured after the supplemental sludge was added in the pilot-scale reactor. (S2–S4 was the same.)

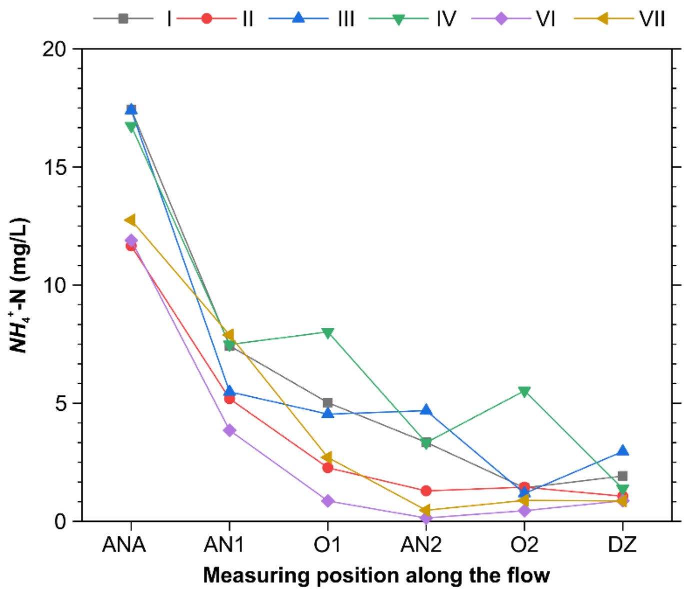

Supplementary Figure S2 Variation in ammonia nitrogen concentration along the flow in different phases.

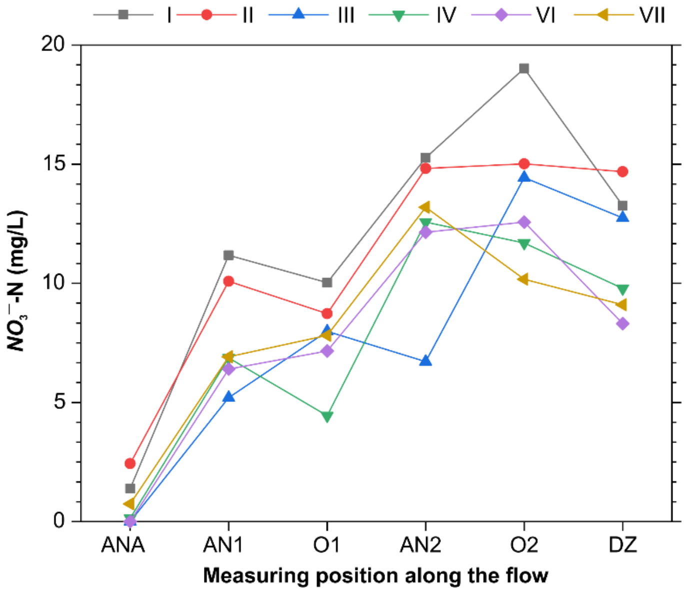

Supplementary Figure S3 Variation in nitrate nitrogen concentration along the flow in different phases.

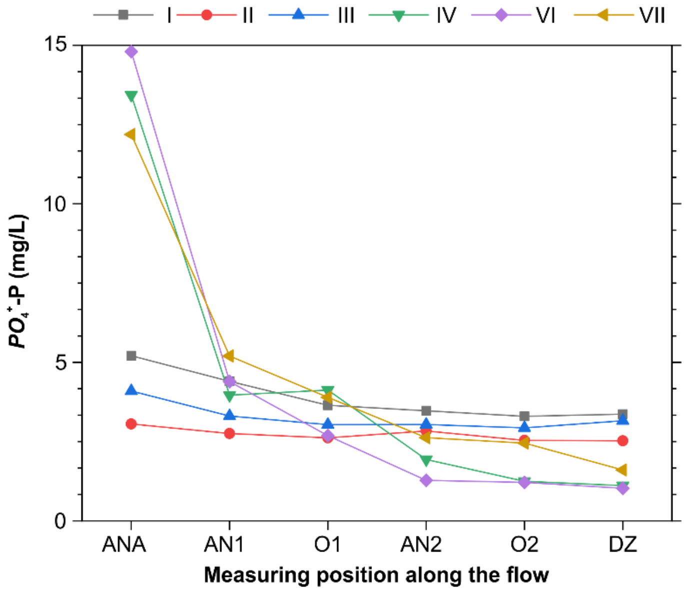

Supplementary Figure S4 Variation in phosphate concentration along the flow in different phases.

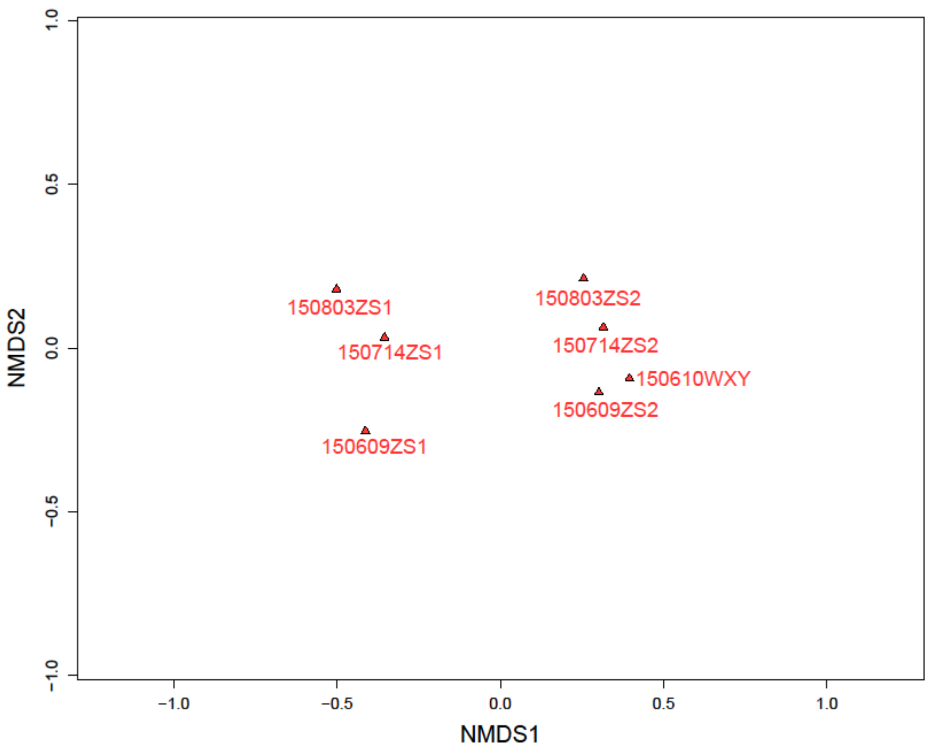

Supplementary Figure S5 Community profiles of the samples based on nonmetric multidimensional scaling(NMDS)
